# Immunohistochemical Analysis of 4-HNE, NGAL, and HO-1 Tissue Expression after Apocynin Treatment and HBO Preconditioning in Postischemic Acute Kidney Injury Induced in Spontaneously Hypertensive Rats

**DOI:** 10.3390/antiox10081163

**Published:** 2021-07-22

**Authors:** Sanjin Kovacevic, Milan Ivanov, Maja Zivotic, Predrag Brkic, Zoran Miloradovic, Rada Jeremic, Nevena Mihailovic-Stanojevic, Una Jovana Vajic, Danijela Karanovic, Djurdjica Jovovic, Jelena Nesovic Ostojic

**Affiliations:** 1 Department of Pathophysiology, Medical Faculty, University of Belgrade, 11000 Belgrade, Serbia; sanjin.kovacevic@med.bg.ac.rs; 2 Department of Cardiovascular Physiology, Institute for Medical Research, University of Belgrade, 11129 Belgrade, Serbia; ivmilan@imi.bg.ac.rs (M.I.); zokim@imi.bg.ac.rs (Z.M.); nevena@imi.bg.ac.rs (N.M.-S.); unajovana@imi.bg.ac.rs (U.J.V.); danijela.karanovic@imi.bg.ac.rs (D.K.); djurdjica@imi.bg.ac.rs (D.J.); 3 Institute of Pathology, Medical Faculty, University of Belgrade, 11000 Belgrade, Serbia; majajoker@gmail.com; 4 Department of Medical Physiology, Medical Faculty, University of Belgrade, 11000 Belgrade, Serbia; wubrkic@yahoo.com (P.B.); rdjeremic@yahoo.com (R.J.)

**Keywords:** acute kidney injury, oxidative stress, 4-hydroxynonenal, neutrophil gelatinase-associated lipocalin, heme oxygenase-1, apocynin, hyperbaric oxygen preconditioning

## Abstract

Oxidative stress has been considered as a central aggravating factor in the development of postischemic acute kidney injury (AKI). The aim of this study was to perform the immunohistochemical analysis of 4-hydroxynonenal (4-HNE), neutrophil gelatinase-associated lipocalin (NGAL), and heme oxygenase-1 (HO-1) tissue expression after apocynin (APO) treatment and hyperbaric oxygenation (HBO) preconditioning, applied as single or combined protocol, in postischemic acute kidney injury induced in spontaneously hypertensive rats (SHR). Twenty-four hours before AKI induction, HBO preconditioning was carried out by exposing to pure oxygen (2.026 bar) twice a day, for 60 min in two consecutive days. Acute kidney injury was induced by removal of the right kidney while the left renal artery was occluded for 45 min by atraumatic clamp. Apocynin was applied in a dose of 40 mg/kg body weight, intravenously, 5 min before reperfusion. We showed increased 4-HNE renal expression in postischemic AKI compared to Sham-operated (SHAM) group. Apocynin treatment, with or without HBO preconditioning, improved creatinine and phosphate clearances, in postischemic AKI. This improvement in renal function was accompanied with decreased 4-HNE, while HO-1 kidney expression restored close to the control group level. NGAL renal expression was also decreased after apocynin treatment, and HBO preconditioning, with or without APO treatment. Considering our results, we can say that 4-HNE tissue expression can be used as a marker of oxidative stress in postischemic AKI. On the other hand, apocynin treatment and HBO preconditioning reduced oxidative damage, and this protective effect might be expected even in experimental hypertensive condition.

## 1. Introduction

The main causes of acute kidney injury are linked to ischemia and hypoxia. Decreased renal blood flow is followed by reduced nutrient and oxygen uptake, which provokes acute tubular necrosis and induces inflammation [1]. Additionally, oxidative stress has been considered as a central aggravating factor in the development of kidney damage and impairment of tissue integrity, leading to renal dysfunction [2,3]. Excessive accumulation of reactive oxygen species (ROS) determines deleterious effects on biomolecules DNA, RNA, proteins, lipids, enzymes, and so on. These changed biomolecules could be detected and used for oxidative stress [4]. ROS may also be produced from nicotinamide adenine dinucleotide phosphate (NADPH) and nicotinamide adenine dinucleotide (NADH) by various oxidase enzymes which are induced by an inflammatory response [5]. Besides ROS, which are inevitable by-products of oxidative stress, there are secondary intermediates such as 4-hydroxynonenal (4-HNE). 4-HNE is a product of lipid peroxidation and may serve as a non-invasive biomarker of oxidative stress [6,7]. Furthermore, 4-HNE is identified as one of the most potent reactive aldehydes [8,9]. In addition to its multiple physiological processes, 4-HNE has been associated with different diseases, such as Alzheimer’s disease, Parkinson’s disease, heart disease, atherosclerosis, cancers, diabetes, and acute lung injury [10,11,12,13,14].

Apocynin (APO, 4-hydroxy-3-methoxyacetophenone) is known as an inhibitor of NADPH oxidase. The precise mechanism of APO activity is not completely understood, but its antioxidant role and anti-inflammatory effects have been shown in many experimental models [15,16]. One of the suggested mechanisms involves activation by myeloperoxidase, as there are data revealing that myeloperoxidase release enhances efficacy of APO, while inhibition of NADPH oxidase is absent in cells deficient of myeloperoxidase [16].

Hyperbaric oxygen therapy (HBO) involves breathing pure oxygen in a pressurized environment. HBO is established as a treatment for many clinical conditions [17,18,19]. At the same time, beneficial effects of HBO preconditioning in postischemic reperfusion injury, were supported by experimental studies and clinical observations [18,19,20]. Alongside increasing the amount of oxygen our blood can carry, HBO therapy can provoke a “controlled” level of oxidative stress, accompanied by an increased level of antioxidants [21]. This rise in oxidative products which do not overcome the antioxidant capacity of the body can trigger different molecular pathways by using the reactive oxygen species as signaling agents [21].

There is much evidence that neutrophil gelatinase-associated lipocalin (NGAL) is an AKI-specific biomarker that can be measured in plasma and urine [22,23], but also is expressed in nephrons during damage development [24]. The prevalence of acute kidney injury has been increasing, but the treatment is only limited to dialysis or transplantation [25,26]. On the other hand, there are encouraging results from the inhibition of oxidative stress and hyperbaric oxygenation preconditioning treatment in ischemic acute kidney injury, induced in animal models [27]. Up to date, all the mechanisms by which HBO achieves the protective and beneficial effects are still unknown. It is reasonable to assume that translating the use of HBO from experimental ischemic conditions into current practice requires better understanding of the mechanisms involved in these HBO positive effects [20]. Heme oxygenase-1 (HO-1) is an inducible isoform of HO with important antioxidant, anti-inflammatory, antiapoptotic, and antiproliferative effects in vascular cells. It is thought to play a key role in maintaining antioxidant/oxidant homeostasis and in the prevention against vascular injury [28]. Because of these cytoprotective effects of HO-1, many drugs have been used in experimental studies to increase HO-1 expression and its activity [29]. Beneficial effects of HO-1 expression might involve different mechanisms. HO-1 catalyzes the breakdown of heme to bilirubin, CO and iron [28]. Heme is a functional component of several intracellular and extracellular proteins that take part in cellular metabolism [30], but at an increased level, heme is a severe prooxidant and harmful stimulus that amplifies oxidative damage in several models of injury [30]. During ischemic injury, heme proteins, which are ubiquitous in cells, destabilize, leading to a significant increase in free heme [30], which would induce HO-1. On the other hand, CO and bile pigments decrease oxidative stress by inhibiting NADPH oxidase and sequestering ROS [30,31,32]. CO upregulates the antioxidant machinery, including superoxide dismutase (SOD), heat shock protein 70 (Hsp70), and activation of nuclear factor-erythroid factor 2-related factor 2 (Nrf2). Iron released from the reaction is safety sequestered by ferritin and thereby mitigates ROS generation [30].

Taking all these mentioned things together, the aim of this study was to perform the immunohistochemical analysis of 4-HNE, NGAL, and HO-1 tissue expression after apocynin treatment and hyperbaric oxygenation preconditioning, applied as single or combined protocol, in postischemic acute kidney injury, induced in spontaneously hypertensive rats (SHR). We also evaluated the effects of these protocols on glomerular filtration rate (by measuring creatinine, urea, and phosphate clearances), as well as on the values of systolic and diastolic blood pressure. We use SHR, because besides the high prevalence of hypertension [33] in population, it might contribute to the increased incidence of AKI [34].

## 2. Materials and Methods

### 2.1. Ethics Statement

The experimental protocol was approved by the Ethic Committee of the Institute for Medical Research, University of Belgrade, Serbia (No. 323-0702569/2018-05/2), in accordance with the National Law on Animal Welfare (“Službeni Glasnik” no. 41/09) that is in line with guidelines for animal research and principles of the European Convention for the Protection of Vertebrate Animals Used for Experimental and Other Purposes (Official Daily N. L 358/1-358/6, 18 December 1986) and Directive on the protection of animals used for scientific purposes (Directive 2010/63/EU of the European Parliament and of the Council, 22 September 2010).

### 2.2. Animals

In this study, we used male SHR (descendants of breeders originally obtained through Taconic Farms, Germantown, NY, USA) at 24 weeks old and about 300 g in weight. The animals were bred at the Institute for Medical Research, University of Belgrade (Belgrade, Serbia), and housed under controlled laboratory conditions (constant temperature 22 ± 1 °C, humidity of 65 ± 1%, 12 h light/dark cycle). The animals were kept in groups of four rats per cage and fed with a standard chow for laboratory rats (Veterinarski zavod, Subotica, Serbia). All animals were allowed with free access to food and water.

### 2.3. Experimental Design

Hypertension was confirmed in all rats by indirect measurement on tail artery (Narco Bio Systems INC, Houston, TX, USA). The animals were divided randomly into five experimental groups: sham-operated rats (SHAM, *n* = 9); rats with induced postischemic AKI (AKI, *n* = 11); animals with AKI and apocynin treatment (AKI + APO, *n* = 11) in a dose of 40 mg/kg body weight, intravenously, 5 min before reperfusion; group exposed to HBO preconditioning before AKI induction (AKI + HBO, *n* = 14) and animals exposed to HBO preconditioning before and treated with apocynin after AKI induction (AKI + APO + HBO, *n* = 9) according to the same protocol as in the AKI + APO group.

Animals with HBO preconditioning were placed into custom-made experimental HBO chamber (Holywell Neopren, Belgrade, Serbia) where were exposed to 100% oxygen according to the following protocol: 10 min of slow compression, 60 min at 2.0 atmospheres absolute (ATA), and 10 min of slow decompression. This protocol was performed twice a day, at 12 h interval, during a two-day period and 24 h before AKI induction. Upon reaching the desired pressure, the flow of oxygen was reduced to maintain constant pressure while allowing the flow out of the chamber. In order to reduce the accumulation of CO_2_ in the chamber environment, this constant exchange was accompanied by a tray of calcium carbonate crystals. This protocol corresponds to a standard hyperbaric oxygen treatment that is routinely used in the clinical setting of Center for Hyperbaric Medicine, Belgrade, Serbia [35], and is in accordance with recommendations of The Committee of the Undersea and Hyperbaric Medical Society [36]. To exclude any confounding issues associated with the changes in biological rhythm, each exposure was started at the same hour. Body temperature was not changed significantly after the HBO preconditioning. AKI induction was performed 12 h after the last HBO preconditioning.

**Figure 1:**
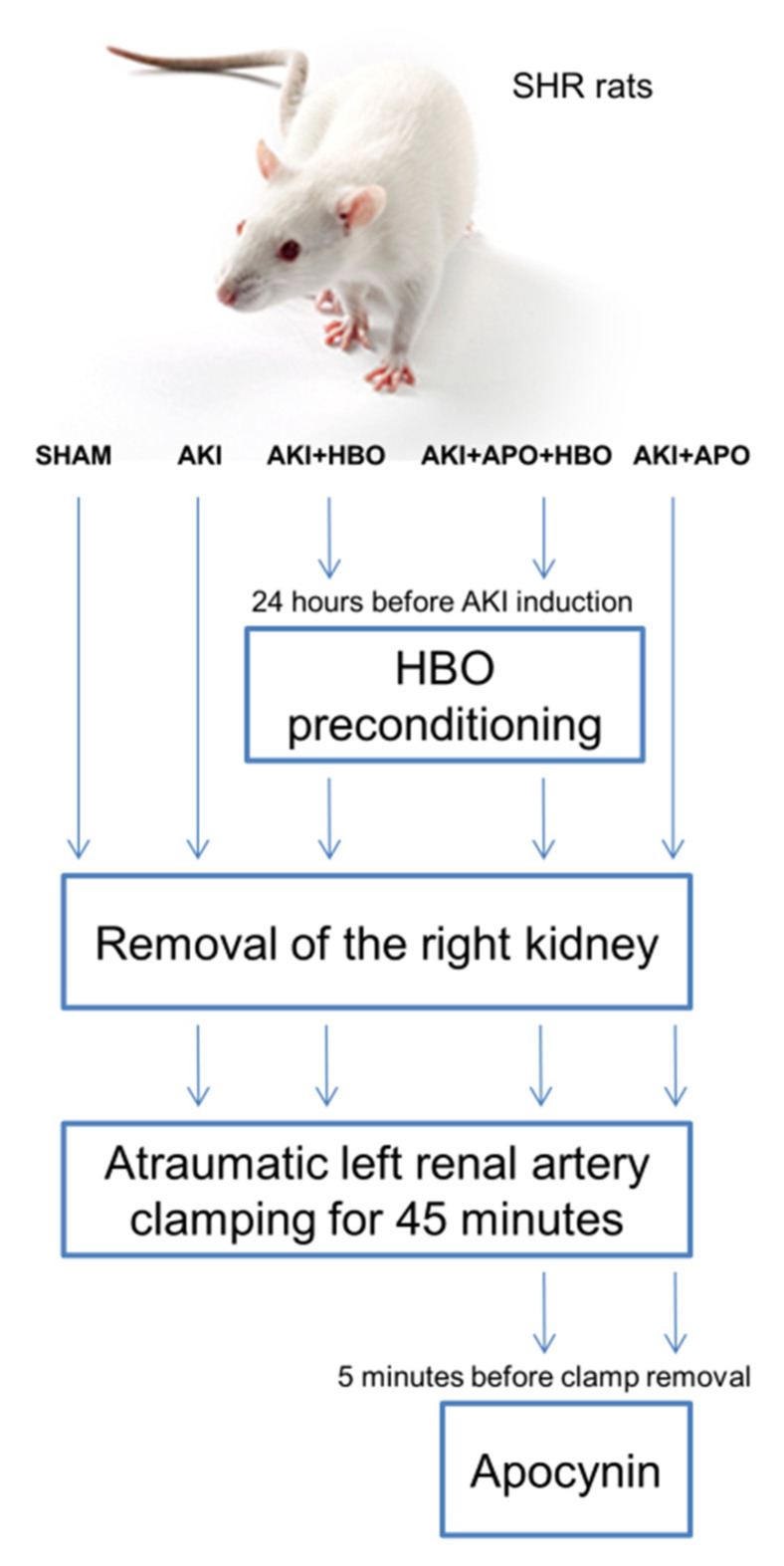
Scheme of the experimental setting. HBO—hyperbaric oxygenation, SHAM—sham-operated rats; AKI—rats with induced postischemic acute kidney injury; AKI + APO—animals with acute kidney injury and apocynin treatment; AKI + HBO—group exposed to HBO preconditioning before acute kidney injury induction; AKI + APO + HBO—animals exposed to HBO preconditioning before and treated with apocynin after acute kidney injury induction.

All surgical procedures were carried out in anaesthetized rats by injecting 35 mg/kg body weight sodium pentobarbital intraperitoneally. During AKI induction, the right kidney was removed while the left renal artery was occluded for 45 min by atraumatic clamp. Identical surgical procedure was performed in SHAM-operated group but without left renal artery clamping. NADPH oxidase inhibitor, Apocynin (Sigma Aldrich, St. Louis, United States of America), was dissolved in hot water, and applied as a bolus injection, intravenously, 5 min before clamp removal, intravenously. Animals without apocynin treatment received bolus of vehicle (saline, 50 μL, intravenously). The scheme of experimental setting is shown in Figure 1.

At the end of AKI induction, the wound abdominal incision was sutured and rats were moved back into metabolic cages for 24 h, with free access to food and water. 

### 2.4. Haemodynamic Measurements

Systolic arterial pressure (SAP) and diastolic arterial pressure (DAP) were measured before all surgical procedures and 24 h after reperfusion, by a direct method, through a femoral artery catheter (PE-50, Clay-Adams, Parsippany, New York, United State of America), connected to a physiological data acquisition system (9800TCR Cardiomax III-TCR, Columbus, Ohio, United States of America), as previously described [37].

### 2.5. Sample Collection

Urine samples were collected before hemodynamic measurements. After hemodynamic measurements, abdominal aorta was punctured to obtain blood samples for further analysis. Blood samples were collected into tubes containing lithium-heparin (Li-heparin, Sigma-Aldrich, St. Louis, MO, USA) and the blood was centrifuged to separate plasma from erythrocytes. Plasma samples were stored at −20 °C until assaying. Kidney tissue was removed immediately after sacrificing and then prepared for histological examination.

### 2.6. Glomerular Filtration

All biochemical parameters for the estimation of glomerular filtration rate (GFR), as a marker of kidney function, were measured using the automatic COBAS INTEGRA 400 plus (Hoffmann-La Roche, Mannheim, Germany) analyser. Creatinine (C_Cr_), urea (C_U_) and phosphate (C_Phos_) clearances were calculated according to standard formula and normalized to body weight. 

### 2.7. Immunohistochemical Analysis 

Immunohistochemistry was applied on formalin-fixed paraffin-embedded kidney samples. Four-micrometer-thick paraffin sections proceeded to deparaffinization and hydratation steps, and afterwards introduced to heat-induced antigen retrieval in citrate buffer (pH 6.0). Novolink™ Polymer Detection System components (Leica Biosystems, Wetzlar, Germany) were applied for immunohistochemistry protocol. Peroxidase and protein blocks were applied prior incubation with primary antibody for 1 h at room temperature. The following primary antibodies were used: 4-HNE (Anti-4 Hydroxynonenal, Abcam, ab46545, 1:100), NGAL (Neutrophil Gelatinase-Associated Lipocalin/Lipocalin-2, R&D Systems, Minneapolis, United States of America, AF1757, 15 μg/mL), HO-1 (Heme Oxygenase 1/HMOX1/HSP32, Novus Biologicals, Littleton, CO, USA, NBP1-31341, 1:100). Secondary antibodies for 4-HNE and HO-1 were applied from Novolink™ Polymer Detection System Kit (Leica Biosystems, Wetzlar, Germany), while NGAL was incubated with rabbit anti-goat IgG (H+L) secondary antibody (Novus Biologicals, Littleton, United States of America, NBP1-74829, 1:1000). Visualization of antigen–antibody reaction by 3,3’-diaminobenzidine (DAB) was used for 4-HNE and NGAL (brown products), while 3-amino-9-ethylcarbazole (AEC) was applied for HO-1 antibody (red products). Subsequent counterstaining with hematoxylin were conducted. Negative controls were performed by omitting the primary antibodies applying incubation with phosphate-buffered saline (PBS) instead. Slides were evaluated using the light microscope BX53 with DP70 camera (Olympus, Hamburg, Germany). The evaluation was performed by two independent pathologists, blinded to the experimental information.

For 4-HNE and NGAL immunohistochemical scoring, according to the intensity and extent of expression in affected kidney structure, following parameters were semiquantitatively evaluated: intensity of expression, on the scale from 0 to 3 (0—without expression, 1—weak expression, 2—moderate expression, 3—strong expression; extent of expression same on scale from 0 to 3 (0—without expression, 1—focal expression, 2—focal to diffuse expression, 3—diffuse expression). For HO-1 immunohistochemical scoring, due to the presence of a different expression pattern, semiquantitative evaluation on the scale from 1 to 6 was performed as following: 1—diffuse and weak expression on the apical surface of the proximal tubular cells, 2—diffuse and moderate expression on the apical surface of the proximal tubular cells, 3—diffuse and weak expression in the cytoplasm and apical surface of the proximal tubular epithelial cells, 4—diffuse and moderate expression in the cytoplasm and apical surface of the proximal tubular epithelial cells, 5—diffuse and moderate expression in the cytoplasm of the proximal tubular epithelial cells, 6—diffuse and strong expression in the cytoplasm of the proximal tubular epithelial cells. The sum of these changes represented the immunohistochemical scores for each parameter and they were used for comparison between groups.

### 2.8. Statistical Analysis

All data are expressed as the mean ± standard deviation (SD). A statistical analysis of each parameter of interest was carried out using analysis Student’s *t*-test for dependent and independent samples and analysis of variance (one–way ANOVA). We used Student’s *t*-test for independent samples to compare animals in AKI group to sham-operated rats. A *p*-value < 0.05 was considered significant. Animals in APO, HBO, and APO + HBO groups were compared to AKI using one–way ANOVA. When a significant F-value in one–way ANOVA test (*p* < 0.05) was obtained, a post hoc test (Dunnett’s multiple comparisons test) was used. Student’s t-test for dependent samples was used to compare SAP and DAP values, before and after surgical procedures, in each group. All statistical calculations were performed using GraphPad Prism for Windows (Version 7.0, GraphPad Software, La Jolla, CA, USA). 

## 3. Results

### 3.1. Hemodynamic Parameters

**Figure 2:**
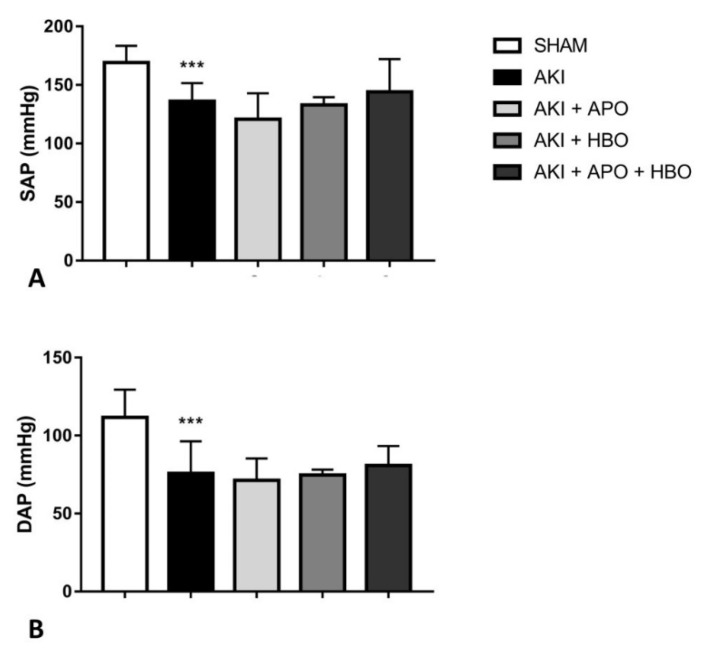
Systemic hemodynamic parameters 24 h after reperfusion; SAP—systolic arterial pressure (**A**), DAP—diastolic arterial pressure (**B**); SHAM—sham-operated rats; AKI—rats with induced postischemic acute kidney injury; AKI + APO—animals with acute kidney injury and apocynin treatment; AKI + HBO—group exposed to HBO preconditioning before acute kidney injury induction; AKI + APO + HBO—animals exposed to HBO preconditioning before and treated with apocynin after acute kidney injury induction. Student’s *t*-test for independent samples (SHAM vs. AKI), one–way ANOVA with Dunnett’s multiple comparisons post hoc test (AKI vs. AKI + APO, AKI + HBO, AKI + APO + HBO); *** *p* < 0.001 vs. SHAM group.

Before surgical procedures, significant changes in SAP and DAP values were not noticed among the groups. On the other hand, 24 h after reperfusion, SAP (*p* < 0.001) and DAP (*p* < 0.001) were significantly decreased in AKI group compared to SHAM group, but without significant differences in AKI + APO, AKI + HBO, and AKI + APO + HBO compared to AKI group (Figure 2).

**Figure 3:**
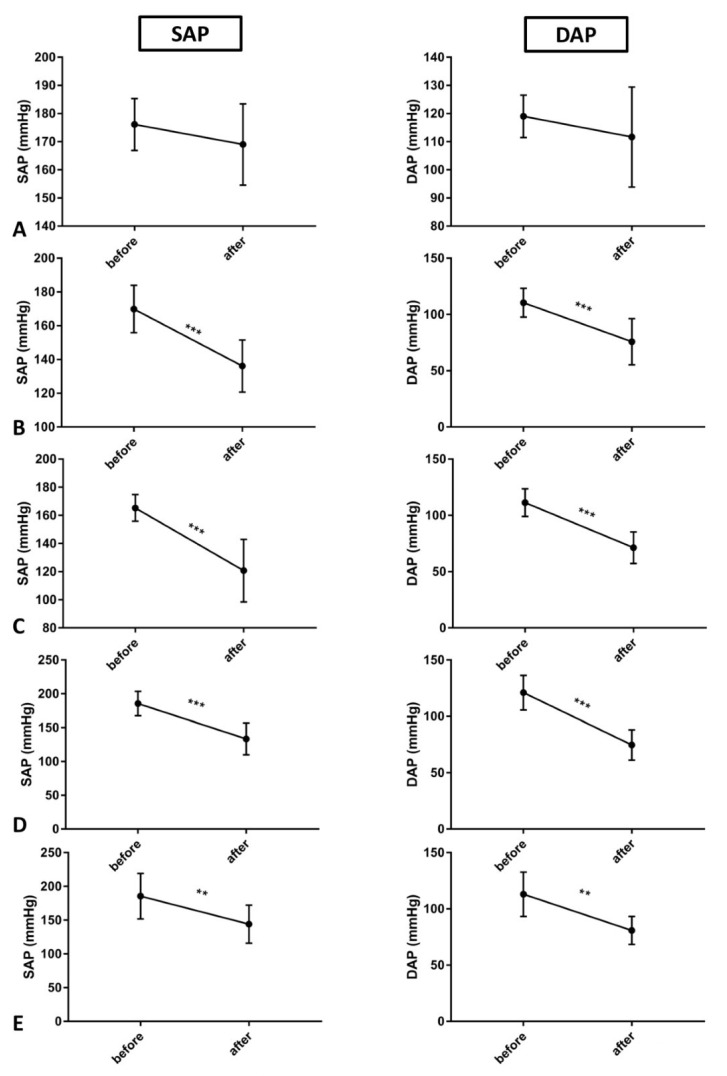
SAP and DAP values before and after surgical procedures in each experimental groups: SHAM (**A**), AKI (**B**), AKI + APO (**C**), AKI + HBO (**D**), AKI + APO + HBO (**E**); SAP—systolic arterial pressure, DAP—diastolic arterial pressure; SHAM—sham-operated rats; AKI—rats with induced postischemic acute kidney injury; AKI + APO—animals with acute kidney injury and apocynin treatment; AKI + HBO—group exposed to HBO preconditioning before acute kidney injury induction; AKI + APO + HBO—animals exposed to HBO preconditioning before and treated with apocynin after acute kidney injury induction. Student’s t-test for dependent samples (before vs. after in each group); ** *p* < 0.01, *** *p* < 0.001.

Considering SAP and DAP values within each experimental group, before and after surgical procedures, SAP and DAP were significantly decreased in all groups in which AKI procedure was performed (AKI, AKI + APO, AKI + HBO, AKI + APO + HBO) (Figure 3). 

### 3.2. Glomerular Filtration Rate

Glomerular filtration rates estimated by creatinine (C_Cr_), urea (C_U_) and phosphate (C_Phos_) clearances are shown in Table 1. AKI induction significantly decreased C_Cr_, C_U_ and C_Phos_ when compared to SHAM group. Remarkable increase in C_Cr_ levels were observed in groups with APO treatment (*p* < 0.01), HBO preconditioning and APO treatment (*p* < 0.05), and HBO preconditioning solitary (*p* < 0.05). Furthermore, remarkable improvement in C_U_ was observed in groups with APO treatment (AKI + APO, *p* < 0.01, AKI + APO + HBO, *p* < 0.01). Considering C_Phos_, significant increase was noticed in AKI + APO (*p* < 0.001), AKI + HBO (*p* < 0.01), and AKI + APO + HBO (*p* < 0.05) in comparison to AKI group.

### 3.3. Immunohistochemical Analysis

#### 3.3.1. 4-Hydroxy-2-Nonenal (4-HNE) Expression

**Figure 4:**
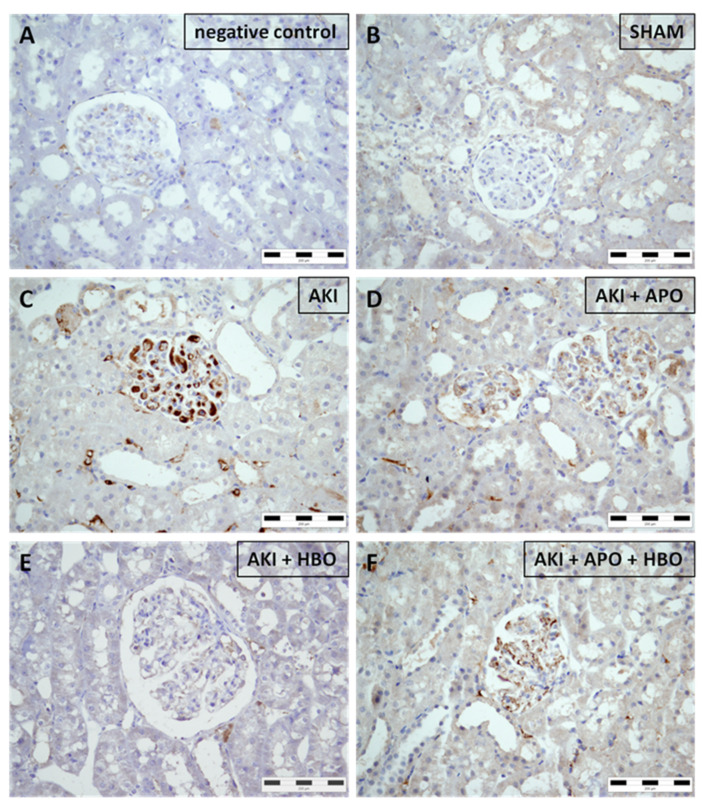
Immunohistochemical 4-hydroxynonenal (4-HNE) expression in representative kidney samples collected in different experimental groups (×400 magnification): negative control (**A**), SHAM (**B**), AKI (**C**), AKI + APO (**D**), AKI + HBO (**E**), AKI + APO + HBO (**F**). SHAM—sham-operated rats; AKI—rats with induced postischemic acute kidney injury; AKI + APO—animals with acute kidney injury and apocynin treatment; AKI + HBO—group exposed to HBO preconditioning before acute kidney injury induction; AKI + APO + HBO—animals exposed to HBO preconditioning before and treated with apocynin after acute kidney injury induction.

Sham-operated rats (Figure 4B) did not express 4-HNE in any parenchymal structure. Nevertheless, it was noticed that AKI induced abundant and strong glomerular expression of 4-HNE along with expression in interstitial compartment (Figure 4C). All treatments significantly decreased 4-HNE expression both in glomeruli and interstitium (Figure 4D–F), with the best results obtained after isolated HBO preconditioning (Figure 4E).

#### 3.3.2. Neutrophil Gelatinase-Associated Lipocalin (NGAL) Expression

**Figure 5:**
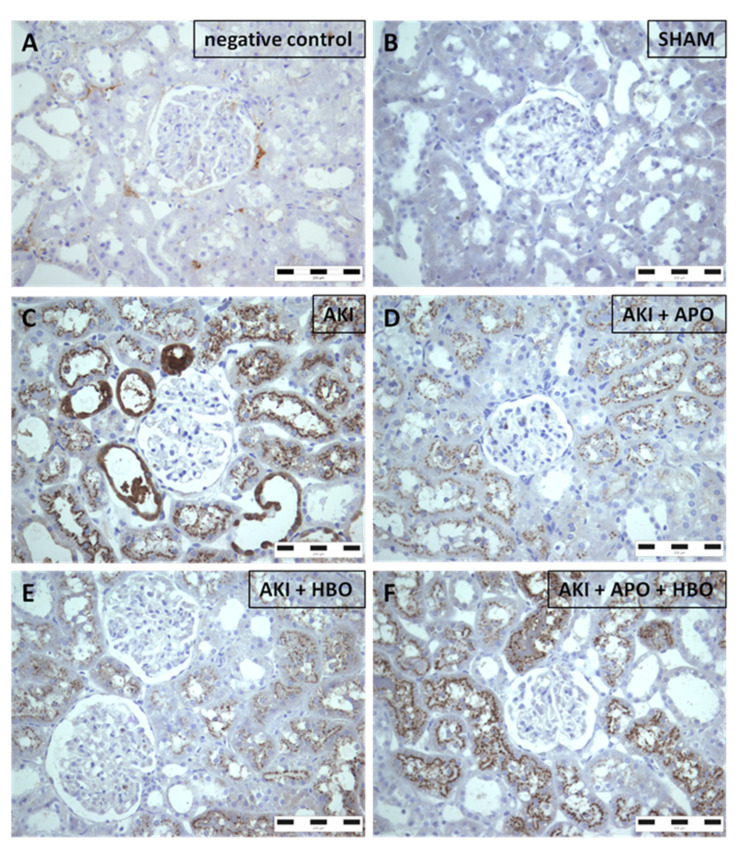
Immunohistochemical neutrophil gelatinase-associated lipocalin (NGAL) expression in representative kidney samples collected in different experimental groups (×400 magnification): negative control (**A**), SHAM (**B**), AKI (**C**), AKI + APO (**D**), AKI + HBO (**E**), AKI + APO + HBO (**F**). SHAM—sham-operated rats; AKI—rats with induced postischemic acute kidney injury; AKI + APO—animals with acute kidney injury and apocynin treatment; AKI + HBO—group exposed to HBO preconditioning before acute kidney injury induction; AKI + APO + HBO—animals exposed to HBO preconditioning before and treated with apocynin after acute kidney injury induction.

Similarly, NGAL was not observed in sham-operated rats (Figure 5B). However, AKI stimulated widespread NGAL expression exclusively in renal epithelial tubular cells with fine granular appearance on the apical surface of the cells, affecting entire circumference of tubular cross-sectioning. This expression pattern was detected in all tubules, including those without morphological evidence of damage. Moreover, NGAL was observed in epithelial cells of apparently dilated tubules (morphological substrate of AKI) (Figure 5C). Treatment with APO (Figure 5D), as well as HBO preconditioning (Figure 5E) and their combination (Figure 5F), reduced NGAL expression and prevented its expression in diffuse and strong pattern in dilated tubules, while preserving slightly reduced granular stain at luminal part of tubular epithelial cells.

#### 3.3.3. Heme Oxygenase-1 (HO-1) Expression

**Figure 6:**
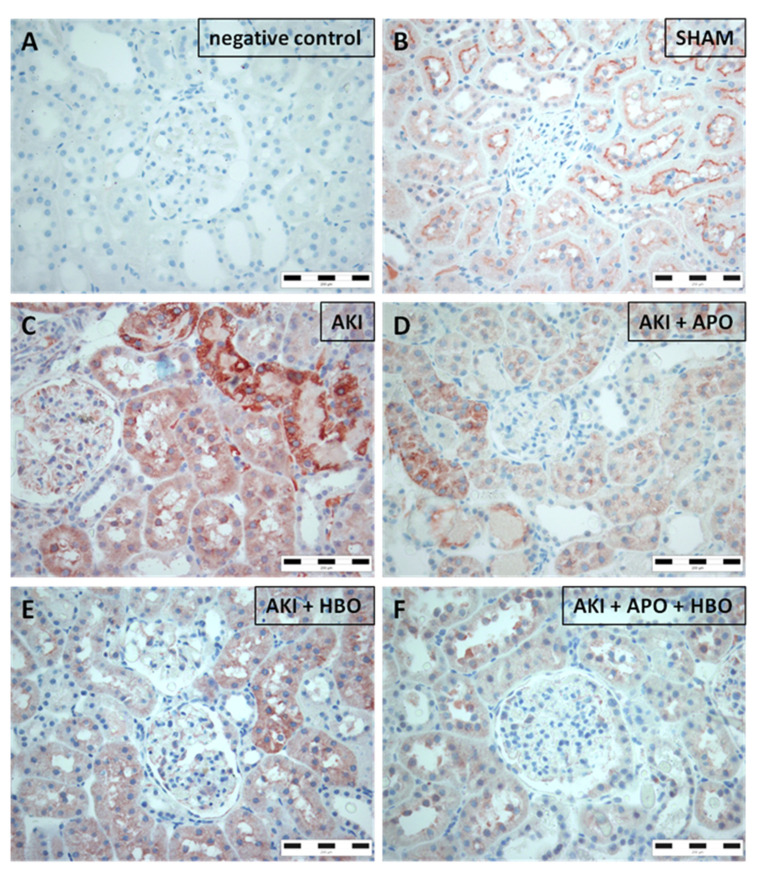
Immunohistochemical heme-oxygenase-1 (HO-1) expression in representative kidney samples collected in different experimental groups (×400 magnification): negative control (**A**), SHAM (**B**), AKI (**C**), AKI + APO (**D**), AKI + HBO (**E**), AKI + APO + HBO (**F**). SHAM—sham-operated rats; AKI—rats with induced postischemic acute kidney injury; AKI + APO—animals with acute kidney injury and apocynin treatment; AKI + HBO—group exposed to HBO preconditioning before acute kidney injury induction; AKI + APO + HBO—animals exposed to HBO preconditioning before and treated with apocynin after acute kidney injury induction.

In the SHAM group, the expression of HO–1 was diffuse with weak-to-moderate intensity on the apical surface of the proximal tubular cells (Figure 6B). In AKI group (Figure 6C), the expression pattern was different, with moderate diffuse expression, but in the cytoplasm of the proximal tubular epithelial cells, with strong expression in some tubules. In comparison to AKI group, in AKI + APO (Figure 6D), AKI + HBO (Figure 6E), and AKI + APO + HBO (Figure 6F) groups the intensity of HO-1 expression was diffuse, but with weak intensity in the cytoplasm of the proximal tubular epithelial cells with also restored expression on the apical surface of the proximal epithelial tubular cells, similar to the expression pattern previously noticed in SHAM group.

#### 3.3.4. Immunohistochemical Score

**Figure 7:**
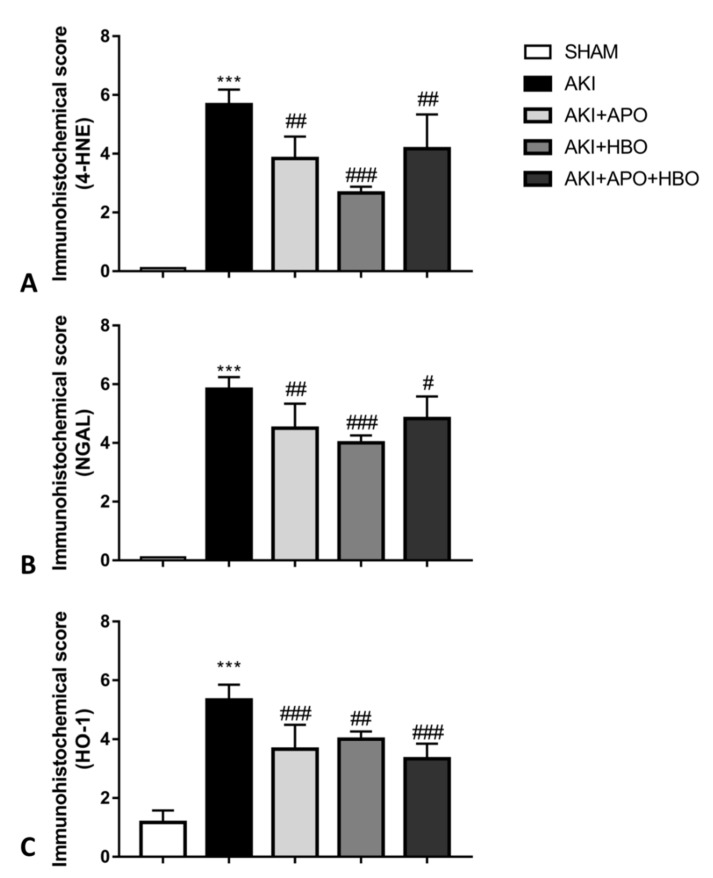
Immunohistochemical score of 4-hydrononenal (4-HNE) (**A**), neutrophil gelatinase-associated lipocalin (NGAL) (**B**) and heme-oxygenase-1 (HO-1) (**C**) expression; SHAM—sham-operated rats; AKI—rats with induced postischemic acute kidney injury; AKI + APO—animals with acute kidney injury and apocynin treatment; AKI + HBO—group exposed to HBO preconditioning before acute kidney injury induction; AKI + APO + HBO—animals exposed to HBO preconditioning before and treated with apocynin after acute kidney injury induction. Student’s *t*-test for independent samples (SHAM vs. AKI), one–way ANOVA with Dunnett’s multiple comparisons post hoc test (AKI vs. AKI + APO, AKI + HBO, AKI + APO + HBO); *** *p* < 0.001 vs. SHAM group; ^#^ *p* < 0.05, ^##^ *p* < 0.01; ^###^ *p* < 0.001.

Immunohistochemical score of 4-HNE, NGAL, and HO-1 expression, as a semi quantitative analysis, was significantly higher in the AKI group for all observed parameters, compared to SHAM control (4-HNE, *p* < 0.001; NGAL, *p* < 0.001; HO-1, *p* < 0.001). By comparison with the AKI group, 4-HNE, NGAL, and HO-1 immunohistochemical score was significantly lower in all treated groups: AKI + APO (4-HNE, *p* < 0.01; NGAL, *p* < 0.01; HO-1, *p* < 0.001), AKI + HBO (4-HNE, *p* < 0.001; NGAL, *p* < 0.001; HO-1, *p* < 0.01) and AKI + APO + HBO (4-HNE, *p* < 0.01; NGAL, *p* < 0.05; HO-1, *p* < 0.001) (Figure 7).

## 4. Discussion

In this study, we showed for the first time that 4-HNE can be used as a marker of oxidative stress in postischemic acute kidney injury induced in hypertensive rats, and that APO treatment, as well as HBO preconditioning, decreased the expression of 4-HNE, in kidney tissue. In the AKI group, we presented, by immunohistochemical analysis, differential expression of 4-HNE in renal tissue. 4-HNE was expressed in glomerulus and interstitium, but there was no expression in proximal and distal tubules. This result can be explained by different 4-HNE degrading capacity of glomerular, mesangial, and tubular cells [38]. Petras et al. determined the degradation of 4-HNE in primary and mesangial cells. They showed that mesangial cells were more susceptible to the toxic effects of 4-HNE. In fact, they presented that the decline of the exogenously added aldehyde 4-HNE was comparable in both cell types, after addition of 1 to 10 μmol/L 4-HNE, but after addition of 100 μmol/L 4-HNE, a drastically lower 4-HNE degrading capacity was found in mesangial cells, as compared to tubular cells [38]. Thus, we can assume that low ability of mesangial and glomerulal cells to degradate 4-HNE may be a potential factor of toxicity of free radicals on the kidney. 4-HNE can form covalent bonds with three different amino acyl side chains, i.e., lysyl, hystidyl, and cysteinyl residues. Due to its amphiphilic nature, the hydroxy aldehyde can diffuse across membranes and covalently modify proteins in the cytoplasm and nucleus, far from their site of origin [39].

The results in this study demonstrated that APO treatment and HBO preconditioning, single or combined, improved creatinine and phosphate clearances in postischemic AKI induced in SHR. The urea clearances were also ameliorated in all treated groups except AKI + HBO. This beneficial effect of APO and HBO on renal function might be explained by different mechanisms. In ischemia-reperfusion injury, the reperfusion phase is the moment when the most harmful effects occur. The initial event during reperfusion is a sudden increase in superoxide anion (O_2_^−^) production in the mitochondria, which is released inside the cell, and O_2_^−^ represents the main trigger for the tissue damage that follows reperfusion [40,41]. Additionally, ischemia-reperfusion injury is followed by inflammatory response, which consists mainly of neutrophils, which generate ROS and are recruited by ROS [42]. Moreover, neutrophils upon activation produce large amounts of O_2_^−^ and ensuing ROS. The enzyme responsible for O_2_^−^ production is NADPH oxidase [43]. As apocynin is experimentally used as an inhibitor of vascular NADPH oxidase, and is known as an antioxidant [44], it is reasonable to assume that apocynin treatment will reduce the level of superoxide anion and improve tissue integrity. Many antioxidant enzymes maintain an appropriate level of ROS and prevent oxidative damages [45]. Additionally, HBO preconditioning may ameliorate creatinine and phosphate clearances in postischemic AKI by upregulation of antioxidant enzymes activities [46,47]. Previously, we showed that apocynin treatment and hyperbaric oxygen preconditioning, with or without apocynin treatment, decreased renal vascular resistance and increase renal blood flow [37], which may further contribute to the improvement of renal function.

Evaluating the results of systolic and diastolic arterial pressure (SAP and DAP), we obtained decreased values of both pressures after AKI induction. This observation was in an agreement with Bowmer study [47]. In fact, the authors of this study have indicated a high uremia influence on diminishing α_1_ adrenoreceptors sensitivity with the cause of reduced mean arterial pressure. This is also consistent with decreased urea clearance in our study. Neither apocynin, nor HBO preconditioning provoke significant changes in SAP and DAP values compared to AKI group. Apocynin can reverse vascular function by reduced ROS production, as well as an increase in NO expression and activity [48], and prevent or reduced pressure elevation. Nevertheless, further investigations are needed to elucidate the possible mechanisms underlying the observed decreased values of SAP and DAP in this experimental model of postischemic AKI. 

Creatinine and blood urea nitrogen clearances, as well as creatinine and urea nitrogen plasma levels are commonly used to estimate renal function [49]. However, numerous studies describe NGAL as a better early marker of acute kidney injury because it is rapidly released after tubular damage [22,23,50]. In animals, ischemia-reperfusion injury of kidneys increases plasma NGAL levels within 3 h after ischemia, whereas serum creatinine level is only moderately increased at the same time [51]. In this study, we demonstrated increased expression of NGAL in tubular cells in the animals that were affected by postischemic AKI. Additionally, apocynin treatment and HBO preconditioning, single or combined, decreased the NGAL tubular expression, which should be considered as beneficial effect of these protocols on the course of induced postischemic AKI. These results are in a line with improved histological and morphological parameters of renal tissues obtained after APO treatment and HBO preconditioning in previous studies [47,52]. 

NGAL’s precise function is still poorly understood. There are growing evidences which point out an important role for NGAL beyond that of a biomarker of renal dysfunction [49]. There are data that NGAL upregulates endogenous antioxidants such as SOD_1_ and SOD_2_ as well as heme oxygenase (HO-1) levels [53,54]. The chemotactic role of NGAL, its role in differentiation and proliferation, and as a growth factor are also showed [49]. In addition, NGAL plays a protective role in AKI after an episode of ischemia/reperfusion [55,56]. In fact, NGAL takes part in iron binding and modulation [57,58]. At the time of ischemia, during an ischemia/reperfusion episode, a large amount of iron-stored is released and provokes oxidative stress, followed by renal damage. Besides this, upcoming reperfusion, further exacerbates tissue impairment caused by oxidative stress [59,60]. 

In physiological conditions, HO-1 protein levels are undetectable, in kidney tissue, except in the tubules, where it is detectable but at low levels [61]. We found low diffuse citoplasmatic expression in proximal tubules in the SHAM group, which can be explained by the fact that we used SHR, and hypertension itself is accompanied with increased oxidative stress [62]. Renal transcription of HO-1 is up-regulated in many stress conditions, including oxidative stress, heat shock, hypoxia, heat shock, toxins, and heavy metals [63]. In AKI, we presented high expression of HO-1 in proximal tubules, which is in accordance with the fact that postischemic AKI is accompanied with hypoxia and oxidative stress [2,3]. The proximal tubules are the cells in the kidney which show the greatest capacity for the overexpression of HO-1 [64]. At the same time, proximal tubular epithelial cells have been shown to be especially sensitive to oxidative stress in vitro [61]. Our results are also in agreement with the findings that HO-1 expression is absent in glomeruli [65]. In this study, in the apocynin treated group, and HBO preconditioning, with or without APO treatment, HO-1 kidney expression was restored close to the control group level. At the same time, apocynin treatment and HBO preconditioning improved oxidative damage in postischemic AKI, which we evaluated by decreased 4-HNE and NGAL tissue expression. On the other hand, amelioration of oxidative stress and improvement in tissue integrity might be the potential reason for decreased induction of HO-1 in treated groups, compared to AKI. 

## 5. Conclusions

In this study, we showed, for the first time, by immunohistochemical analysis, that 4-HNE can be used as a marker of oxidative stress in postischemic acute kidney injury animal model. We also showed that apocynin treatment, with or without HBO preconditioning, improves creatinine and phosphate clearances in postischemic AKI induced in SHR. This improvement in renal function was accompanied with decreased 4-HNE, while HO-1 kidney expression was restored close to the control group level. NGAL renal expression also decreased after apocynin treatment and HBO preconditioning, with or without APO treatment. Considering our results, we can say that apocynin treatment, as well as HBO preconditioning, reduced oxidative damage induced in postischemic AKI, and these protective effects might be expected even in experimental hypertensive condition. These results are encouraging and pave the way for further research with the aim that one day they may be applied in clinical settings.

## Figures and Tables

**Table 1:** Creatinine (C_Cr_), urea (C_U_) and phosphate (C_Phos_) clearances 24 h after reperfusion.

Title	C_Cr_ (mL/min/kg)	C_u_ (mL/min/kg)	C_Phos_ (mL/min/kg)
SHAM	4.03 ± 1.68	1.88 ± 0.89	0.71 ± 0.40
AKI	0.30 ± 0.18 ***	0.09 ± 0.03 ***	0.22 ± 0.10 **
AKI + APO AKI + HBO AKI + APO + HBO	1.70 ± 0.98 ^##^ 1.33 ± 1.25 ^#^ 1.44 ± 0.077 ^#^	0.38 ± 0.21 ^##^ 0.27 ± 0.22 0.30 ± 0.18 ^#^	0.62 ± 0.16 ^###^ 0.54 ± 0.32 ^##^ 0.48 ± 0.20 ^#^

C_cr_—creatinine clearance, C_U_—urea clearance, C_Phos_—phosphate clearance; SHAM—sham-operated rats; AKI—rats with induced postischemic acute kidney injury; AKI + APO—animals with acute kidney injury and apocynin treatment; AKI + HBO—group exposed to HBO preconditioning before acute kidney injury induction; AKI + APO + HBO—animals exposed to HBO preconditioning before and treated with apocynin after acute kidney injury induction. Student’s *t*-test for independent samples (SHAM vs. AKI), One–way ANOVA with Dunnett’s multiple comparisons post hoc test (AKI vs. AKI + APO, AKI + HBO, AKI + APO + HBO); ** *p* < 0.01, *** *p* < 0.001 vs. SHAM group; ^#^ *p* < 0.05, ^##^ *p* < 0.01, ^###^ *p* < 0.001 vs. AKI group.

## Data Availability

The data presented in this study are available in article.
